# Supplementary material for: Are carbon emissions trading and green financial instruments synergistic? -Comprehensive quantitative research based on content analysis
Source: PLoS One. 2024 Mar 7;19(3):e0298601. doi: 10.1371/journal.pone.0298601 (PMC10919601; doi:10.1371/journal.pone.0298601)
Supplement: S1 Data — (ZIP) [file pone.0298601.s001.zip › Data/地级市Z_F工作报告.zip!/╣π╢1⁄2/║╙╘┤╩╨/└┤╫╘╣1⁄2╓┌║┼í╛┬φ┐╦╩2╛▌═°í┐_2014.docx]

各位代表：

现在，我代表市人民政府向大会作政府工作报告，请予审议，并请市政协各位委员和其他列席人员提出意见。

2013年工作回顾

2013年，是我市贯彻党的十八大精神的开局之年，是我市攻坚克难、重点突破的进取之年。市政府在省委、省政府和市委的正确领导下，在市人大及其常委会和市政协的监督支持下，突出“项目推进年”、“园区提升年”和“民生建设年”，以稳中求进、好中求快为总基调，以提高经济增长质量和效益为中心，求真务实，开拓进取，较好完成了市六届人大三次会议确定的目标任务，经济社会和政府建设呈现七大喜人变化。

——增长速度变快。坚持发展为第一要务，攻坚克难，敢谋善成，全市经济提速发展。预计全市生产总值686.89亿元，比上年增长12.1%，提速0.5个百分点；人均生产总值22706元，增长11%。规模以上工业总产值和增加值分别为1265.6亿元和285.2亿元，分别增长16.9%和17%。地方公共财政预算收入48.77亿元，增长29.58%，提速9.58个百分点。固定资产投资334.3亿元，增长20%。社会消费品零售总额236.59亿元，增长13%。实际利用外商直接投资2.09亿美元，增长6%；外贸出口21.17亿美元，增长8.4%。城镇居民人均可支配收入18502元，增长12%；农民人均纯收入8782元，增长13%。民营经济占全市生产总值比重达60%。节能降耗和污染减排任务顺利完成。

——发展空间变宽。坚持园区为第一平台，大力推动园区扩容提质增效，促进三大产业平稳较快发展。 工业园区建设卓有成效。“一区六园”开发建设投入10亿元，扩容3.65平方公里，配套设施不断完善，接纳能力进一步提升；实现工业增加值175亿元、增长30%，占全市工业增加值比重64%；实现税收18亿元、增长30%，占全市税收比重23%。深圳（河源）和盐田（东源）转移园获评省优，罗湖（源城）转移园被省评为“四星级优秀服务园区”，龙岗（紫金）转移园升级为省级转移园，南山（龙川）转移园成功复牌，园区经济呈现蓬勃生机。严格土地监管，集约节约用地水平不断提升，争取省下达我市新增建设用地指标16523亩、增长43.8%，保障了园区发展用地需求。 农业园区建设稳步推进。积极推进灯塔盆地国家现代农业示范区建设，获得国家、省扶持资金近2亿元。新增市级以上农业龙头企业15家、农民专业合作社600家。新认证无公害农产品9个、绿色食品3个、有机农产品1个。高标准基本农田建设扎实推进，耕地保护工作荣获省一等奖。农林牧渔总产值134.79亿元，增长5.5%。 生态旅游园区加快建设。万绿生态旅游产业园成功竞得3亿元省扶持资金。第十届旅游文化节取得圆满成功。巴伐利亚养生庄园、康泉十八国际生态健康旅游城等重点旅游项目顺利推进。接待入市游客1905.1万人次、增长15.4%，旅游总收入152.6亿元、增长15.2%。 金融业加快发展。政银企合作不断深化，金融机构向中小微企业贷款余额273.5亿元，占各项贷款余额的47.54%；新型金融机构加快发展，河源成为省农村金融改革创新综合试点。

——内生动力变强。坚持项目为第一抓手，着眼于解近忧、谋长远，不断增强经济发展动力和后劲。 “四个一批”项目、“三个50”和“三个100”工程取得突破。“三个50”工程年度任务超额完成，新签约项目80个，合同投资额203.83亿元；新开工项目81个，到位资金38.76亿元；新投产项目68个，到位资金69.97亿元。“三个100”工程成效显著，减产企业减少68家，新增规模以上工业企业90家、限上商贸企业42家，为历年之最。 重点项目建设扎实推进。全市省、市重点项目完成投资141亿元，汉能10MW光伏发电、精电增资扩产等项目顺利投产。龙川大帽山、紫金南岭、和平黎明风电场项目列入国家“十二五”风电项目核准计划，市商业中心、市高新区保税区进口商品交易中心列入省建设规划。 “两园一工程”进展顺利。完成两园土地选址和总体规划编制工作，展开征地拆迁和基础设施建设；成功举办中国新技术矿产产业发展高峰会议暨河源稀土产业招商推介会，合同投资110亿元，绿色资源产业化迈出新步伐。市区水源工程顺利开工建设。

——城乡面貌变美。坚持交通为第一基础，大力推动中心城区扩容提质，不断加强城乡基础设施建设，城乡宜居水平进一步提高。 基础设施建设有新进展。完成交通基础设施投资47.3亿元，增长96.3%。大广、河紫高速公路进展顺利。改造国、省道和县乡公路112公里和108公里。赣龙紫与河惠莞高速公路纳入省规划。完成一批水库除险加固工程和小型灌区改造工程，建成解决农村4.3万人饮水安全工程，小型农田水利重点县和水利示范县项目顺利推进。完成电网建设投资4.2亿元，一批变电站和线路工程建成投产。 中心城区提质扩容取得新突破。江东新区发展总体规划获得省政府常务会议批准，江东新区城市总体规划成果编制完成，启动了起步区征地拆迁和项目布局、基础设施建设工作。市区迎客大桥、大环市路、滨江大道二期、东江西路北段、东江东路河职院段、宝源一路跨铁路桥工程顺利推进，城市框架进一步拉大；完成市区凯丰路、兴源路、客家文化公园西路、长堤中路及亲水步道升级改造，改造一批公交站亭，解决了市区重点区域“水浸街”问题；全民健身广场、客家文化公园二期建成开放，城市功能进一步完善。市农副产品批发中心三鸟市场建成开业，群丰和东城农贸市场竣工使用。 新农村建设有新成果。积极创建名镇名村和“幸福村居”，扎实推进宜居村镇“六个一”工程，完成20个建制镇和500个村庄规划编制，县城生活垃圾无害化填埋场建成或开工建设，完成村道硬底化554.7公里。加快推进山区县农村综合改革，40%乡镇和20%村建立社会综合服务平台，40%乡镇建立农村集体资产管理交易平台。完成农村集体土地所有权确权登记发证和农村土地承包经营确权登记试点工作。

——生态环境变优。坚持生态为第一资源，牢牢守住环保底线，加强生态环境建设，努力实现“绿富双赢”。 环境保护力度加大。新丰江水库代表广东参加全国江河湖泊生态环境保护竞争立项，成功竞得中央5.93亿元扶持资金。环保基础设施建设加快，环万绿湖6镇污水处理设施建成使用，完成白岭头河、黄子洞河截污管网提升改造。全面启动东江水环境整治，落实南粤水更清行动计划，东江流域水浮莲整治成效显著，高埔河、东埔河整治进展顺利，江湖水质持续改善。完成市区环境空气自动监测系统升级改造。强化环保执法监管，开展专项整治行动，完成节能减排任务。 生态建设步伐加快。启动新一轮绿化河源大行动，完成86公里生态景观林带示范段和91.24万亩“一灭三改”森林碳汇重点生态工程建设任务。大力开展严厉打击非法采矿和矿山复绿行动，捣毁非法矿点228个，复绿面积7670亩。加强涉林企业监管，强化森林防火管控，有效打击涉林违法犯罪。东源、和平、紫金县竞得省碳汇造林扶持资金共2.59亿元。

——民生事业变实。坚持保障和改善民生为第一目标，社会事业协调发展，民生不断改善。 教育创强成效明显。新增15个省教育强镇，连平县成功创建省教育强县。全面完成学前教育三年行动计划，学前三年教育毛入园率达87.62%。新建、改造义务教育标准化学校50所，小学和初中学龄入学率达99.99%和99.65%。巩固提升普及高中阶段教育水平，高中阶段毛入学率达89.11%。职业教育加快发展，河职院成为省示范性高职院校立项建设单位，市卫校新校区二期工程稳步推进，市体育运动学校启动建设。建成3所特殊教育学校，“三残”儿童小学入学率提高至98.9%。 社会保障水平提升。新增就业岗位4.8万个、劳动力培训转移就业4.8万人，安置失业人员再就业、就业困难人员再就业1.2万人，城镇登记失业率控制在2.45%。“五大险种”参保率提高，新农合、城镇居民医保参保率分别达100%和96%以上，基本实现人群全覆盖和应保尽保。成功应对超两百年一遇的“8·16”特大洪涝灾害，2276户全倒户今年春节前可住进新房。物价水平保持稳定，新建88家平价商店。残疾人事业稳步发展，源城区、东源县、和平县、龙川县残疾人综合服务中心建成投入使用。 卫生计生服务体系进一步健全。市人民医院、市妇幼保健院二期工程及市精神卫生防治中心工程建设稳步推进。进一步深化医药卫生体制改革，基层综改工作任务基本完成，3个省公立医院改革试点县工作扎实推进；启动市高新区同济医院和东源县人民医院创建平价医院工作；开展医疗质量大检查和打击非法行医专项行动。完成人口计划，继续稳定低生育水平。 “文化河源”加快推进。积极创建全国文明城市，社会文明程度不断提高。加强文物保护利用和非物质文化遗产保护，启动可移动文物普查。开展文化惠民活动，丰富群众文化生活。开展文化市场整治行动，加强新闻出版和广播电视管理。获得国家恐龙遗址保护资金4770万元，河源恐龙博物馆升级为“中国古动物馆恐龙蛋馆”。连平县“忠信花灯节”列入“全国春节文化特色活动”。专利申请量突破1000件，获评全国科技进步考核先进市。完成乡镇（街道）综合文化站评估定级。完成广播电视“户户通”和县区政府机关使用正版软件工作。与韩国安城市缔结为友好城市。 扶贫开发扎实开展。启动新一轮扶贫开发，投入资金5.44亿元，283个贫困村启动扶贫项目1299个，完成农房改造2.2万户，“两不具备”村庄搬迁安置农户2861户。妥善解决水库移民安置点问题，移民生产生活水平稳步提高。 社会治理不断加强。扎实推进平安河源建设，开展公安机关“八大会战”、“四大行动”和“九大雷霆”等专项整治行动，实施社会创新项目和社会管理“四个一”工程，积极开展普法和人民调解，社会治安综合治理和信访维稳工作得到加强。社会组织有序发展，新增146个社会组织。扎实开展“南粤（河源）幸福活动周”活动。全面落实安全生产“一岗双责”制度，安全生产形势总体稳定。产品质量和食品药品安全监管、应急管理等工作得到加强。 “十件实事”实现预期目标。投入民生资金122.38亿元，教育创强、城乡充分就业、改善农村生产生活条件等实事的目标任务超额完成，“四馆两中心”、市人民医院等医疗机构扩建工程、保障性安居工程、提高城乡最低生活保障水平和农村五保供养标准、生活垃圾无害化填埋场和转运站、改善市区交通状况等基本完成预期目标。

——行政效能变高。严格落实中央“八项规定”，认真开展“四风”整顿和作风暗访活动，干部作风继续向好。认真开展“三赛”活动，进一步完善考核机制，加强“亮灯”管理和督查督办，狠抓工作落实，形成了比学赶超和力争上游的良好氛围。调整压减行政审批事项392项、压减率达41.2％；成立市公共资源交易中心；开通省网上办事大厅河源分厅；完成“12345”政府服务热线平台整合升级并运行；开展县镇政府性债务、财政税收、民生项目、国有企业、领导干部经济责任等审计工作，促进了政府服务更加规范、透明、公正和高效运作。人大代表建议、政协提案办复满意和基本满意率100%。

此外，妇女儿童、法律援助、人民武装、民族宗教、“双拥”创建、优抚安置、驻外机构、机关事务、殡葬管理、地方志、人事、对台、老龄、人防、气象、打私等各项工作取得新进展。

各位代表，我们克服了各种困难，实现了加快发展。这是省委、省政府和市委正确领导的结果，是全市人民共同奋斗的结果，也离不开广州、深圳、中山等兄弟市和海内外各界人士的关心支持。在此，我代表市人民政府，向全市广大干部群众、驻河源部队、消防官兵和各民主党派、人民团体、社会各界人士致以崇高的敬意！向关心支持我市改革发展的兄弟市以及港澳同胞、台湾同胞、海外侨胞表示衷心的感谢！

我们也清醒地认识到，我市经济社会发展还存在不少困难和问题。主要表现在：工业提质增效还不够实，工业园区接纳能力不足，新引进重大项目和新投产项目相对较少；城市综合竞争力还不够强，中心城区扩容提质力度有待加大；绿色资源转化还不够快，生态保护和节能减排任务更加艰巨；有些部门及干部抓落实的方法还不够多，力度不大、成效不明显，等等。对此，我们必须高度重视，认真加以解决。

2014年工作安排

2014年是深入贯彻党的十八届三中全会精神，全面深化改革和实现跨越发展的关键之年。我们既面临挑战，更面临难得的历史机遇，必须振奋精神、凝心聚力、突出重点、狠抓落实，牢牢把握工作主动权。

今年政府工作的指导思想是：深入贯彻党的十八届三中全会、中央经济工作会议、省委十一届三次全会精神，全面落实市委六届五次全会决策部署，继续坚持稳中求进、好中求快的总基调，紧扣“三个抓手”，突出转型升级、提质扩容、“三农”问题、交通建设、深化改革、生态文明、改善民生七大着力点，既求速度、更重质量效益，全面推进河源跨越发展，为打造“广东绿谷”、建设幸福河源奠定良好基础。

今年全市经济社会发展的主要预期目标是：生产总值增长12.5%；全社会工业增加值增长18%；地方公共财政预算收入增长20%；固定资产投资增长20%；社会消费品零售总额增长12%；外贸进出口总额增长3%；居民消费价格指数涨幅控制在3.5%以内；城镇登记失业率控制在3%以内；城镇居民人均可支配收入增长12%；农村居民人均纯收入增长13%；人口自然增长率控制在13.4‰以下；完成节能减排任务。

为实现上述目标，必须正确处理好四大关系： 一是正确处理好加快发展与转型升级的关系。解决河源的问题，归根到底要靠加快发展。必须在加快发展中转型升级，抓住粤东西北地区振兴发展的战略机遇期，充分发挥投资的关键作用，大力培育服务业新的增长点，以“用地少、效益高、创新多、能耗低、生态好”为发展导向，推动发展方式实质性转变。 二是正确处理好加快发展与环境保护的关系。坚持“点上发展、面上保护”。既要依托优势资源，做好做活“水头”、“石头”和“木头”文章，更要坚守作为广东生态屏障和重要饮用水源地的政治使命和生态责任，保护好、弘扬好客家古邑、万绿河源特有的山水美、自然美、人文美，为子孙后代留下一个资源丰厚的“绿色银行”，为广东生态文明保有一片碧水蓝天。 三是正确处理好加快发展与深化改革的关系。坚持深化改革是加快发展的重要法宝。深化改革只有进行时，没有完成时，要切实理顺政府与市场、社会的关系，充分发挥好市场在配置资源中的决定性作用，让改革成为加快发展的不竭动力。 四是正确处理好加快发展与改善民生的关系。检验加快发展的成效，最终要看人民是否得到了真正的实惠，民生是否得到了真正的改善。改善民生要突出重点、守住底线，在解决突出问题上下功夫，及时准确了解群众所思、所盼、所忧、所急，把民生工作做实、做深、做细、做透，为加快发展凝心聚力。
